# Computer-aided imaging analysis in acute ischemic stroke – background and clinical applications

**DOI:** 10.1186/s42466-019-0028-y

**Published:** 2019-08-15

**Authors:** Yahia Mokli, Johannes Pfaff, Daniel Pinto dos Santos, Christian Herweh, Simon Nagel

**Affiliations:** 1 Department of Neurology, University Hospital Heidelberg, INF 400, 69120 Heidelberg, Germany; 2 Department of Neuroradiology, University Hospital Heidelberg, Heidelberg, Germany; 3 Department of Radiology, University Hospital Cologne, Cologne, Germany

**Keywords:** Acute ischemic stroke, Imaging, Computer aided diagnosis, Artificial intelligence

## Abstract

Tools for medical image analysis have been developed to reduce the time needed to detect abnormalities and to provide more accurate results. Particularly, tools based on artificial intelligence and machine learning techniques have led to significant improvements in medical imaging interpretation in the last decade. Automatic evaluation of acute ischemic stroke in medical imaging is one of the fields that witnessed a major development. Commercially available products so far aim to identify (and quantify) the ischemic core, the ischemic penumbra, the site of arterial occlusion and the collateral flow but they are not (yet) intended as standalone diagnostic tools. Their use can be complementary; they are intended to support physicians’ interpretation of medical images and hence standardise selection of patients for acute treatment. This review provides an introduction into the field of computer-aided diagnosis and focuses on the automatic analysis of non-contrast-enhanced computed tomography, computed tomography angiography and perfusion imaging. Future studies are necessary that allow the evaluation and comparison of different imaging strategies and post-processing algorithms during the diagnosis process in patients with suspected acute ischemic stroke; which may further facilitate the standardisation of treatment and stroke management.

**Electronic supplementary material:**

The online version of this article (10.1186/s42466-019-0028-y) contains supplementary material, which is available to authorized users.

## Background

The diagnosis of stroke is based on the clinical examination and also on different imaging technics. The differentiation between haemorrhagic and ischemic stroke and the detection of large vessel occlusions (LVO) represent key steps in determining the optimal therapy regimen for the individual patient. Time is essential: the faster the diagnosis is made, and the appropriate therapy is initiated, the better the outcome of patients [1]. Many tools for medical image analysis have been developed to reduce the time needed to detect abnormalities and to provide more accurate results. Particularly, tools based on machine learning techniques have led to significant improvements in medical imaging interpretation in the last decade.

In the 1980s, a concept called computer-aided diagnosis (CAD) was introduced. Its primary intent was to provide radiologists with a second opinion while reading their cases [2]. CAD has seen remarkable developments, and applications for all modalities of medical imaging have been presented. Although CAD systems are widely available, their implementation in clinical routine varies with the clinical scenario in which they are applied. In the first years following the emergent of CAD concept, the majority of the developed algorithms focused on the early detection of breast cancers on mammograms and the detection of lung cancer on chest radiographs or computed tomography. Currently, CAD systems are well established for providing an aid diagnosis in stroke and many other medical fields (Additional file 1).

### CAD concept spectrum

CAD is strongly related to Artificial intelligence (AI), a branch of computer science that has witnessed an incredible development in the last few years. CAD and automated computer diagnosis (ACD) are two concepts with similar names but different meanings.

CAD usually relies on a combination of interpretation of medical images through computational algorithms and the physicians’ evaluation of the medical images. In this case, medical physicians are not replaced by an algorithm, but they use algorithms’ output as a second opinion. The diagnosis and the final decision are made at the end of the process by the physicians [3]. This concept is useful particularly in cases where physicians are less confident about the diagnosis so that the final decision may be improved by the use of algorithms’ results. However, it is worth noting that this approach can have downsides as well, and some studies suggest that users tend to become less vigilant when aware that of the CAD results while interpreting medical images [4].

ACD is based on computer algorithms only, and the diagnosis is made directly by the algorithm. In this case, the algorithm’s performance in a clinical routine must be at least equal or better than the performance of an average physician. Although computer algorithms may easily exceed human performance in many fields, developing such tools for the usage in medical imaging remains a difficult task. More difficult is even the adequate assessment and validation of CAD or ACD based algorithms.

In 2011 Goldenberg et al. presented another concept of CAD for the usage in emergency medicine called computer-aided simple triage (CAST), which performs an analysis of medical images and sorts them into different prioritisation’s categories. CAST systems should attract the radiologists’ attention to acute and time-sensitive critical cases [5].

Physicians use CAD systems as a support for their decision-making process aiming to get better results in the detection and interpretation of pathologies in medical images. Evaluating the performance of CAD schemes against a gold standard is usually not sufficient; measuring the influence of CAD systems on the decisions made by the physicians and on the general workflow in a clinical setting is also crucial. This influence may be positive, and this could be mirrored by a decrease of time need to diagnosis establishment or an increase in the detection rate of true subtle anomalies due to CAD support. A negative influence may be marked by an increase in false positive results due to the confusion generated by the bias effect. Therefore, although not easy, it is essential to study the effect of CAD systems on their users. For such studies, a large number of physicians or radiologists is needed, and a comparison between final results obtained by the physicians with or without getting CAD support in different settings is helpful to determine the impact of these tools on the performance of the physicians, which is related to the outcome of patients.

### Classical and modern CAD algorithms

CAD algorithms are developed to perform tasks that usually require human intelligence and aim at extracting patterns from medical images and using these patterns to perform a specific task like suggesting a diagnosis.

Pattern recognition and extraction requires prior identification of relevant image features. Classical multistep CAD systems are based on conventional machine learning algorithms in which human-experts hand-engineer these features. Classical CADs process data in multi-steps (at least two: hand-encoded features extraction and classification) [6].

Unlike these, modern CAD systems use representation learning (RL) based algorithms, in which no manual feature encoding is necessary [7]. RL schemes determine the best features to use while classifying the input data on their own. Processing steps of modern CAD are sometimes not distinguishable, because of their structural properties usually based on neuronal networks with multiple hidden layers.

**Fig. 1:**
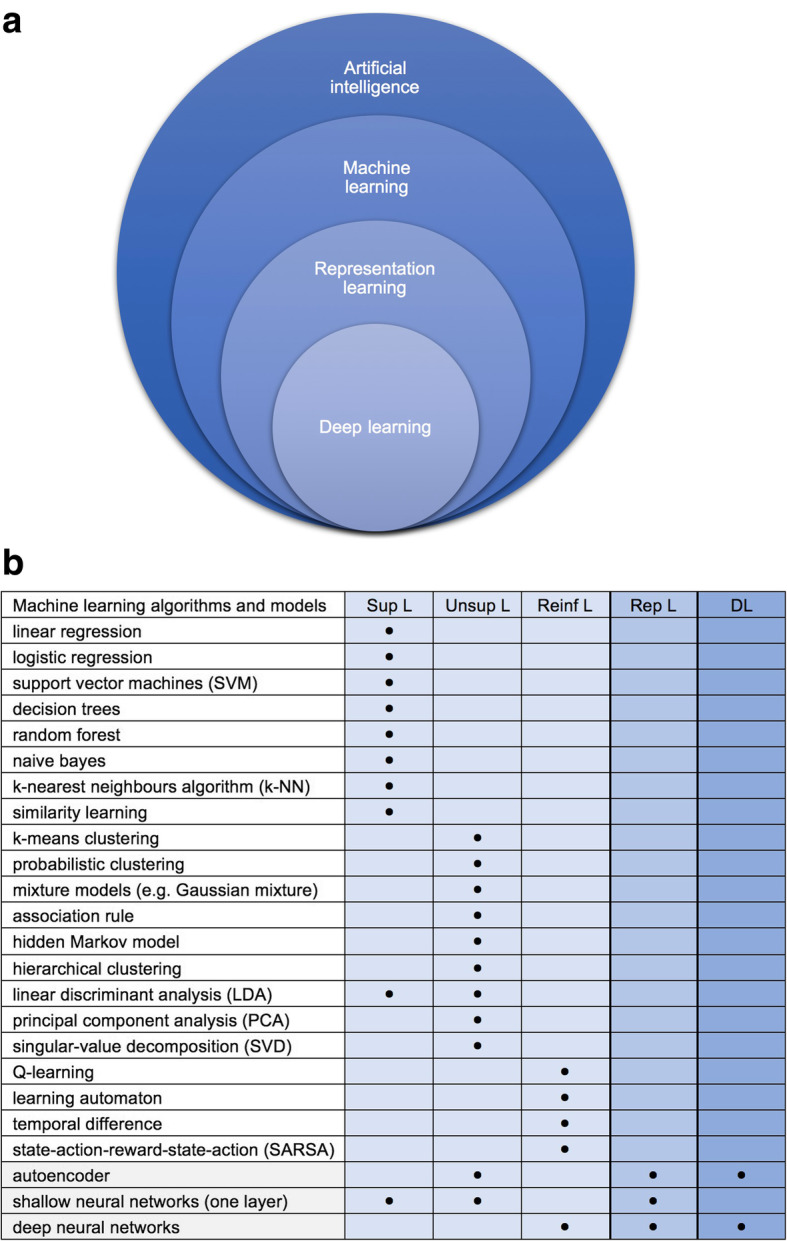
**a** A Venn diagram presenting that deep learning is a type of representation learning, which is a kind of machine learning, which is a subfield of Artificial intelligence (adapted from [8]). **b** A summarised representation of the most common machine learning algorithms and models. Sup L: Supervised learning: labelled data is used. Unsup L: Unsupervised learning: unlabeled data is used. Semi-supervised learning: a mixture of supervised and unsupervised learning. Reinf L: Reinforcement learning: learning by doing (rewarding correct- and punishing wrong actions). Rep L: Representation learning: automatic generation of features. DL: Deep learning: hierarchical representation learning

Deep learning (DL) is a subfield of RL, in which algorithms get from simple features, like edges or textures, to more complex features such as shapes or organs in their learning process (Fig. 1a and b) [8, 9]. Through deep learning astonishing results have been made possible in previously very challenging visual tasks such as the ImageNet challenge. Consequently, these methods have already been successfully applied to the medical field, e.g. for the detection of melanoma [10] and the detection of intracranial haemorrhage [7].

Classical and modern CAD systems alike are usually trained by using labelled data; this method is called supervised learning. The labelling is commonly done by a human expert; the algorithm’s possible output-results in this model are well defined. It is worth noting that this is a crucial step in developing e.g. deep learning-based algorithms. It can generally be said, that a larger dataset for training should lead to a more robust algorithm that would be less prone to overfitting and should be able to perform better on previously unseen external data. However, large datasets are not easy to obtain, and labelling large datasets is time-consuming if done by hand, but prone to errors if done automatically. On the other hand, unsupervised learning algorithms try to discover previously unknown patterns and structures in the unlabelled input data without previous labelling. The algorithm decides by itself how to cluster the data into different subgroups. A combination of these two methods is called semi-supervised learning, in which a large amount of unlabelled data in conjunction with a usually small quantity of labelled data are used [9]; this method could merge benefits of both previously cited approaches (more accuracy as in supervised learning, and less time for data labelling as in unsupervised learning).

### Processing steps of CAD systems

**Fig. 2:**
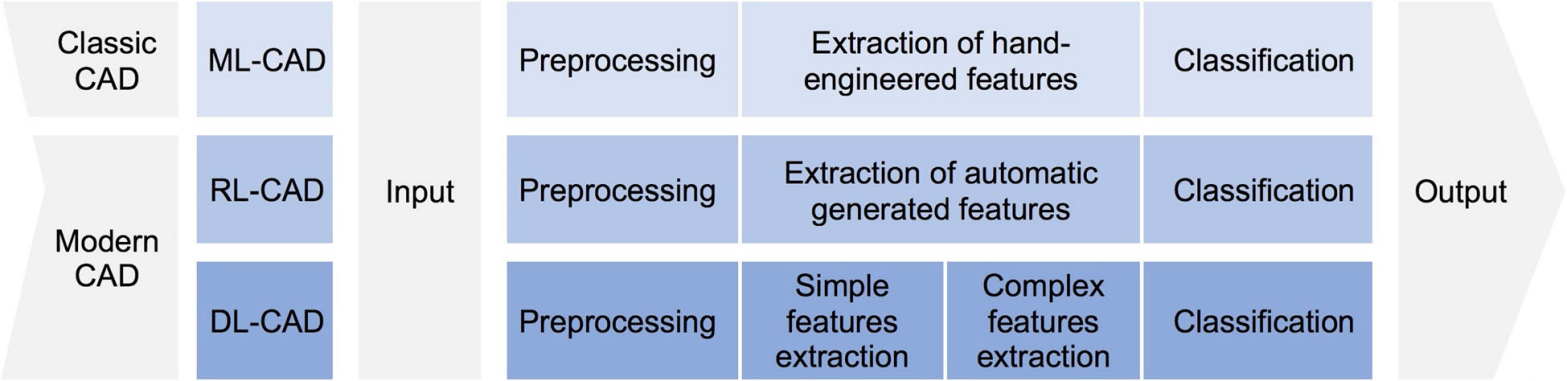
Graphic representation of different processing phases in CAD systems. ML-CAD: Machine learning based computer-aided diagnosis; RL-CAD: representation learning based computer-aided diagnosis; DL-CAD: Deep learning based computer-aided diagnosis. Preprocessing phase is optional. (adapted from [8])

Pattern recognition process in classical and modern CAD medical algorithms usually follows three main steps; however, getting through all these steps is not mandatory. These main steps are: (i) preprocessing of medical images – including segmentation and designation of regions of interest (ROI), (ii) extracting automatically generated or hand-engineered features that are predefined from human experts and finally (iii) data classification based on these features (Fig. 2). Modern CAD algorithms can present output data without necessarily getting through all these steps; this was made possible after the introduction of neural networks with multiple hidden layers.

(i) Preprocessing of medical images is essential to simplify interpretation and the subsequent processes. Different technics can be used, such as image resizing and application of smoothing filters for noise reduction.
(ii) During the feature extraction stage, the algorithms determine the characteristic of objects or ROIs, which can then be used in the classification step. There are many feature extractors and they differ in their processing method, time to extract features and also their computational methods.
(iii) For the final classification task, the CAD system can either perform simple two-class categorisations or more refined multi-class categorisations. The first one would only classify features in the medical images in two categories, for example normal and abnormal. The second one could classify the obtained features into various categories and thus provide differential diagnosis to some extent.

### Commercial CAD applications in stroke field

Especially since the advent of deep learning, medical image analysis using AI is a worldwide rapidly growing market [11]. Several software tools have been made commercially available that aim to support radiologists and medical doctors in making more rapid and precise decisions in the diagnosis of stroke, which could be beneficial for patients’ outcome. Table 1 lists main companies and their presented applications.

**Table 1:** Overview of commercially available software applications for automated and semi-automated medical image analysis for acute stroke diagnostics (Descriptions are based on information provided by the companies on their official websites. Some companies also offer algorithm outside the ischemic stroke field; we listed them for completion but do not further discuss those)

Company	Software	Description
Aidoc	Aidoc Head	triages stroke patients using non-contrast CT scans by flagging suspected intracranial haemorrhages and highlights cases that require immediate attention in worklist
Apollo Medical Imaging Technology	CT Perfusion: Stroke	CTP stroke module is a part of MIStar software package. It generates brain perfusion maps using deconvolution algorithms together with Apollo’s noise reduction and motion artefact correction technologies
	DSC-MRI: Stroke	DSC-MRI perfusion module is a part of MIStar software. It features both parametric curve analysis and deconvolution algorithm for perfusion maps with easy identification of arterial input function
Brainomix	e-ASPECTS	assess the ASPECTS^a^ score and volume of ischemia in non-contrast CT images
	e-CTA	standardizes the assessment of collaterals in CTA scans
inferVISION	AI-CT (head)	gets information about type of stroke (haemorrhagic or ischemic), determines location, volume and severity of haemorrhagic strokes
iSchemaView	RAPID CTA	automatically provides CTA maps and identifies brain regions with reduced blood vessel density
	RAPID CTP	provides cerebral perfusion maps
	RAPID MRI	provides fully automated diffusion and perfusion maps
	RAPID ASPECTS	automatically identifies and scores regions with early ischemic changes using ASPECTS
JLK Inspection	JBS-01 K	Ischemic stroke subtype (TOAST^b^) classification solution based on MR images and clinical information data
	JBS-02 K	Ischemic stroke severity (NIHSS^c^) prediction solution based on MR images, clinical information data and 3D hybrid artificial neural network technology
	JBS-03 K	Ischemic stroke prognosis (3-month mRS^d^) prediction solution based on MR images, clinical information data and 3D hybrid artificial neural network technology
	JBS-04 K	Haemorrhagic stroke detection and classification solution based on CT images and 3D hybrid artificial neural network technology
	JBS-05 K	Hyperacute ischemic stroke detection solution based on CT images and clinical information data
	JBS-06 K	Hyperacute ischemic stroke detection solution based on MRI, clinical information data and 3D hybrid artificial neural network technology
	JBA-01 K	Aneurysm detection solution based on MR angiography, clinical information data and 3D hybrid artificial neural network technology
Max-Q AI	AccipioDx	diagnostic tool that rules out the presence of intracranial haemorrhage in non-contrast CT scans
mbits	mRay-Modul veocore	Perfusion analysis tool
Nico.lab	StrokeViewer	provides analysis of relevant biomarkers from stroke imaging (NCCT, CTA, dynamic CTA and follow-up imaging). The following have been clinically validated: Haemorrhage detection and quantification, thrombus identification and evaluation, collateral assessment, follow-up infarct volume quantification, ASPECTS (in development)
Olea Medical	Olea Sphere	automatically computes core, penumbra and mismatch ratio in CT and MR perfusion images
Qure.ai	qER	detects critical abnormalities such as bleeds, fractures mass effect and midline shift, localizes them and quantifies their severity in head CT
	qQuant	suite of quantification and progression monitoring products for CT and MRI scans (e.g. brain tumour volume)
Viz.ai	Viz LVO	automatically identifies and triages suspected large vessel occlusion (LVO) strokes
	Viz CTP	automatically analyse CT perfusion images
Zebra Medical Vision	AI1	All-In-One (AI1) Application with included algorithm for intracranial haemorrhage detection. AI1 detects also other medical conditions like low bone mineral density, vertebral fractures and more

^a^ ASPECTS: Alberta stroke programme early CT score

^b^ TOAST: Trial of Org 10,172 in Acute Stroke Treatment

^c^ NIHSS: NIH Stroke Scale

^d^ mRS: modified Ranking Scale

Many methods have been applicated to evaluate the performance of CAD systems for commercialization. Some examples of these methods are leave-one-out, cross-validation, hold-out, and resubstitution. However there is until now no standardized approach; trying to solve this issue, the Computer Aided Detection in Diagnostic Imaging Subcommittee (CADSC) which is a committee initiated by the American Association of Physicists in Medicine (AAPM) proposed some recommendations on the methodology applicated in the evaluation of CAD system performance.

Health authorities firmly regulate the commercialisation of medical devices and drugs. Medical softwares are usually included under the medical devices category. Software as a Medical Device (SaMD) is a new term defined by the International Medical Device Regulators Forum (IMDRF) for applications that are used without being a part of a hardware medical device. These SaMD have special regulations and validation processes, which are adopted by the American Food and Drug Administration (FDA). Within the European Economic Area, medical softwares must obtain a certification mark (CE Marking) demonstrating conformity with medical devices regulations (MDR) approved by the European Parliament and Council before free commercialization. Of note, some of these certification processes do not require the presentation of clinical validation data.

## Non-contrast enhanced computed tomography

### Signs of infarction

A non-contrast-enhanced computed tomography (NCCT) brain scan is still the most widely available tool in acute stroke imaging because it is easily accessible, inexpensive, efficient, fast and reliably rules out haemorrhage. The most accurate assessment of the early infarction is obtained by diffusion-weighted magnetic resonance imaging (DWI) [12]. However, DWI is not everywhere available in the acute setting. Quantitative measurements of acute infarct on NCCT are difficult in clinical routine, since signs of infarction are more subtle and human assessment is highly variable. Hence, the correct NCCT interpretation of a patient with an acute ischemic stroke before thrombolysis or thrombectomy requires training and experience.

The probably first semi-automated approach to identify putative hypodensity within the middle cerebral artery (MCA) territory was published in 2001. In the following years, several other different computer-aided detection schemes for cerebral ischemia on CT were published. However, all of these papers described different approaches and focused on the methodology of the algorithms used; sample sizes were rather small and rigorous comparisons against the current gold standard, the interpretation of the scan by a neuroradiologist or against DWI were missing (Additional file 1). Up to date, only two commercial products are available that are certified for use in clinical routine: the e-ASPECTS® software from Brainomix Ltd. (Oxford, UK) and RAPID ASPECTS® by iSchemaView (Menlo Park, USA).

Siemens developed another post-processing tool for early ischemic change detection in CT using the ASPECT score (syngo.via Frontier ASPECT Score Prototype V1_2_0, Siemens Healthcare GmbH, Erlangen, Germany), which is not yet certified for clinical application but has undergone comparison to the e-ASPECTS software [13]. Here the authors found high agreement in ASPECTS rating between two certified radiologists, expert consensus reading of NCCT images, and e-ASPECTS, but only low to moderate agreement to Frontier-ASPECTS by Siemens.

e-ASPECTS, RAPID ASPECTS, as well as Frontier-ASPECTS, are based on quantitative evaluation of early focal ischemic damage by the Alberta Stroke Programme Early CT Score (ASPECTS), which is a topographic scoring system that divides the MCA territory into ten areas of interest. Originally ASPECTS was calculated within two prespecified slices through the level of the basal ganglia and the level of supra-ganglionic structures [14], while softwares are now integrating the whole brain scan and visually highlight the damaged ASPECTS region. e-ASPECTS from version 7 also displays acute ischemic volume in millilitres illustrated by a coloured heat map. The automated assessment overcomes the significant intra- and interrater variability of ASPECTS and hence standardizes the clinical application [15, 16]. Current guidelines recommend ASPECTS as an imaging selection criterion for mechanical thrombectomy (MT) in patients within 6 h from stroke onset [17].

While so far only one study on the performance of RAPID ASPECTS has been published [18], there are several studies on the performance of e-ASPECTS within different settings and patient populations available [19–24]. All these studies indicate that these algorithms can be better than non-stroke experts and at least equal than experts in applying the ASPECTS to patients with acute ischemic stroke, yet they are not intended as a stand-alone diagnostic tool. Furthermore, e-ASPECTS has been compared to CT perfusion with regard to prediction of clinical outcome and final infarct size in patients with large vessel occlusion undergoing MT [25, 26]. The results of both studies suggest that NCCT based infarct cores estimation can be an alternative to computed tomography perfusion (CTP) derived infarct core estimation. Recently it was also shown that e-ASPECTS ratings and further clinical criteria could be successfully used to identify suitable candidates for MT in patients with longer or unknown time windows [27]. Importantly, the only studies showing an increase of physician’s performance before and after the aid of an automated algorithm are available for e-ASPECTS [28] and e-CTA® [29].

### Hyperdense vessel sign

Hyperdense vessel sign (HDVS) on NCCT represents an early marker of acute ischemic stroke caused by intracranial arterial occlusion. HDVS is a radiological phenomenon marked by an increase of vessel radiodensity on NCCT after an acute occlusion. HDVS can be seen in various vascular diseases, including acute arterial occlusion, acute arterial dissection, aneurysm rupture, and acute venous thrombosis [30].

HDVS is most commonly reported in the MCA region; this is because MCA territory is usually the most affected cerebral region by ischemic stroke [31]. Also, the MCA has a large diameter in comparison to other intracerebral arteries and the majority of its branches run parallel to the –commonly most reconstructed– transverse imaging plan in cranial CT.

HDVS is highly specific (95%) and moderately sensitive (55%) for arterial obstruction in acute ischemic stroke; usage of thin-slicing improves the sensitivity significantly [32]. However, physiological calcifications or hyperdense structures outside cranial arteries are frequent. Koo et al. defined some objective criteria of MCA HDVS to differentiate it from normal MCA vessels. These are: In NCCT, the density of the pathological MCA should be superior to 43 Hounsfield units (HU) and 1.2 times higher than the contralateral MCA [33]. Lim et al. published a work about the value of HDVS in detecting large vessel occlusions (LVO) in the setting of neurological acute ischemic presentation, especially for hospitals with no access to CT-Angiography. They concluded that the HDVS has a high sensitivity and specificity for recognizing LVO on thin-slice NCCT in acute ischemic stroke patients presenting with an NIHSS more than 10 and suspected occlusion of MCA (M1 segment) or basilar artery [34].

Through its early visibility, already after vessel occlusion and before upcoming pathological parenchymal ischemic changes, it represents a perfect diagnostic aid in time crucial acute stroke cases. Automating the process of MCA or intracranial HDVS sign detection in emergency imaging may accelerate the identification of positive cases, especially in spoke centers without regular access to CTA. This could improve referral logistics and reduce the time to acute treatment, e. g. mechanical recanalization or systemic thrombolysis. Important success elements of CAD of HDVS are: Acquisition of high-quality cranial native CT, application of new reconstruction methods like iterative model reconstruction to reduce noise and improve diagnostic performance [35] and using thin slicing to improve the sensitivity of HDVS. Studies suggest that potential algorithms can achieve a sensitivity if up to 97,5% in detecting HDVS (Additional file 1). Such software may also help also with the triage and patients selection process for endovascular reperfusion therapy by notifying the medical team within minutes [36].

## CT angiography

### Collateral assessment

Recent trials have demonstrated the therapeutic potential of thrombectomy even in an extended time window [37, 38]. Patients who can be treated successfully in such an extended time window are referred to as “slow progressors” and it is thought that sufficient collateral blood flow is the key essential to this phenomenon [39]. In the acute stroke set-up, CTA can visualise collateral flow independent of particular arterial territories as compared to DSA. There are numerous qualitative and semi-quantitative scales to score collaterals [40] (collateral scores; CS). A common scoring system was established by Tan et al. [41]. Here, the whole MCA territory is graded from 0 (no collaterals visible) to 3 (no difference to the contralateral hemisphere). However, this score does not take into account anatomic location and functionality and can cause considerable inter-observer variability [40]. As an alternative, anatomic regions like those underlying the ASPECTS can be applied to score collaterals [42] which has been shown to increase the concordance between different readers in comparison the NCCT-ASPECTS [43]. The fact that different scores exist and are used in parallel is mainly due to the fact that there is no “ground truth” against which these scores could be validated. Information from DSA examinations, which is the gold standard for depiction of intracranial vessel, cannot be compared immediately. This is because in CTA contrast agent (CA) is given intravenously and spreads systemically throughout the arterial vasculature and can thereby reach the occluded territory in a retrograde fashion.

**Fig. 3:**
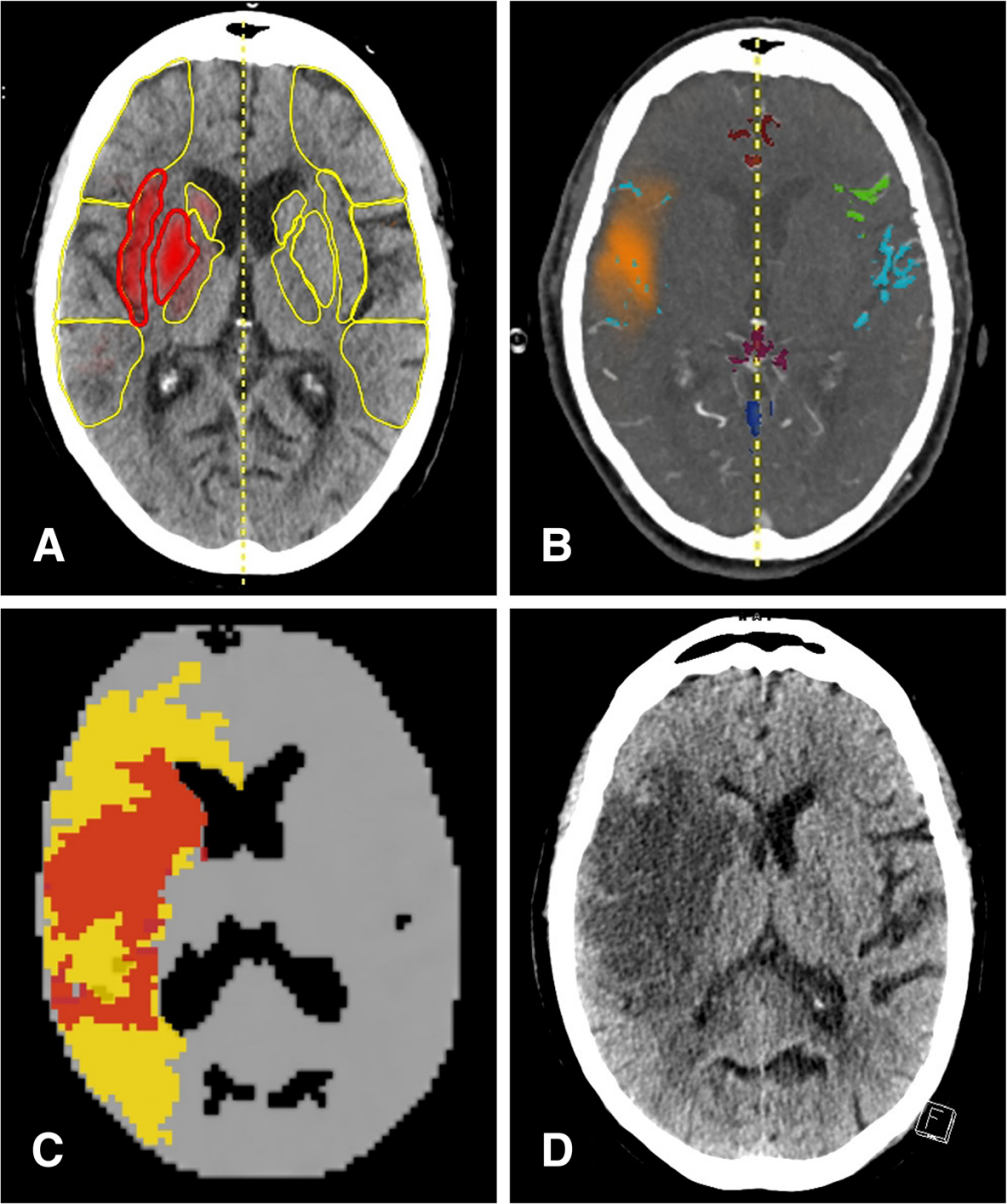
Acute LVO with insufficient collateral flow and extended infarction despite successful recanalization: An 84-year-old woman suffered from an acute hemiparesis (NIHSS 14) due to an M1 right-sided M1-occlusion. e-ASPECTS (**a**) was 8 due to early signs of infarction in the caudate head and lentiform nucleus, e-CTA collateral score (**b**) was 1 (21%), and there was a large area of hypoperfusion with an only moderate mismatch (**c**). Neurological deficit persisted (NIHSS 12) despite full recanalization (mTICI 3) within 5 h from symptom onset and follow up NCCT at 24 h (**d**) shows near complete infarction of the MCA territory. Note the difference of the arterial vessels (**b**, blue colour) compared to the opposite side as well as the reduced parenchymal contrast (**b**, orange cloud) on the affected side

**Fig. 4:**
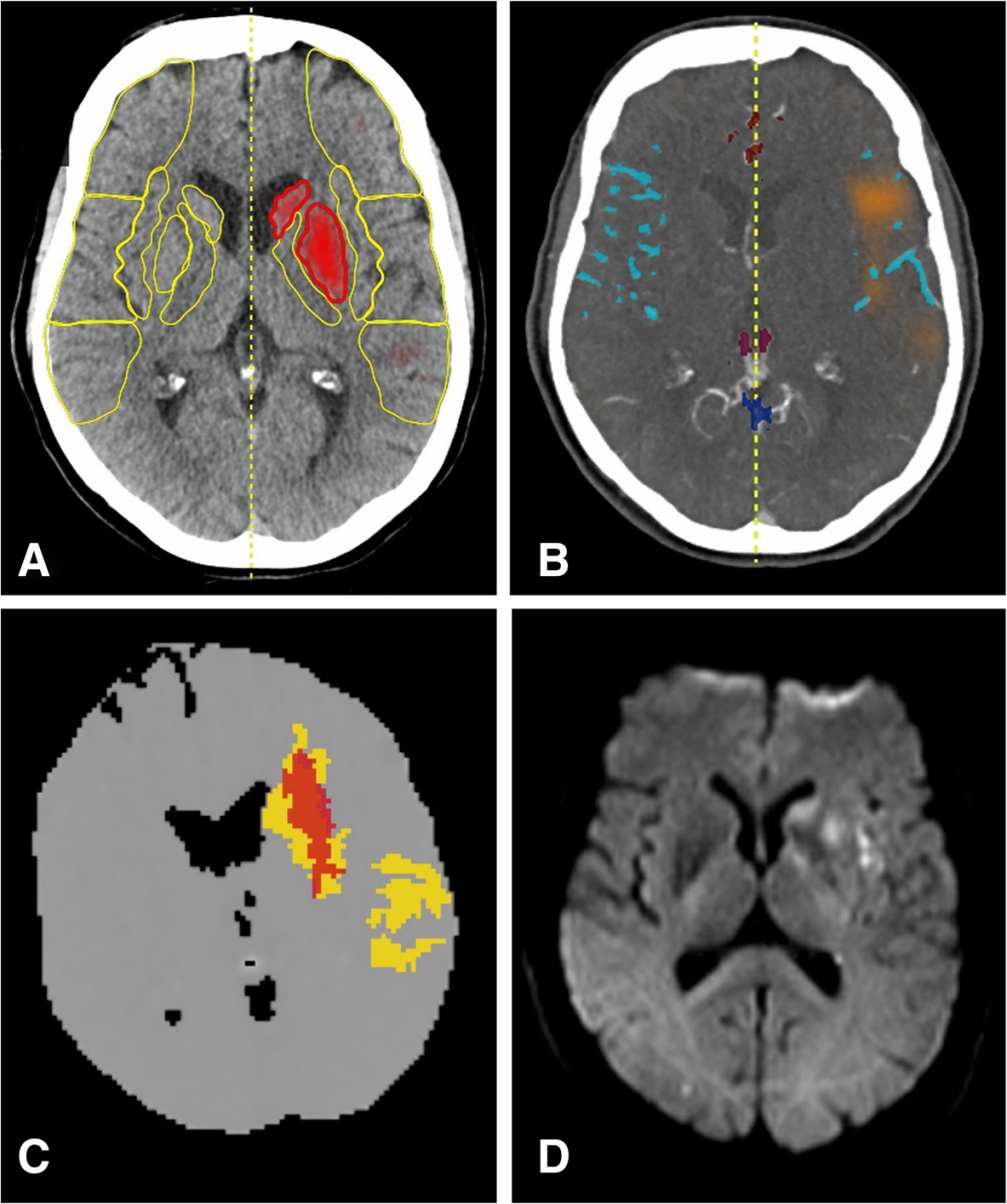
Acute LVO with sufficient collateral flow, successful recanalization and good outcome: A 77-year-old woman suffered from an acute hemiparesis and aphasia (NIHSS 17) due to an left-sided M1 occlusion. As in case 1, e-ASPECTS (**a**) was 8 with the caudate head and lentiform nucleus being affected, but e-CTA collateral score was 2 (54%, **b**), and the hypoperfused area (**c**) is considerably smaller. Again, full recanalization (mTICI 3) could be achieved within 5 h from symptom onset, and the patient recovered completely (NIHSS 0). Follow up MRI at 36 h (**d**) shows incomplete infarction of the striate only

As an alternative, validation can be done indirectly by investigating the ability of a particular score to predict the clinical outcome of the patient, appropriate therapy (i.e. vessel recanalization) provided. Consequently, CTA-based scoring of collaterals has proven to be predictive not only for the success of recanalization but also for clinical outcome in several MT studies [44–46]. Different imaging patterns and patients outcomes dependent on collateral flow are illustrated in Figs. 3 and 4.

It was also demonstrated that the timing of the CTA is crucial for the assessment of collateral flow and that acquisition in the late arterial or early venous phase increases the specificity [47]. This is important since arterial CTA typically aims at the early arterial phase which could lead to an underestimation of collateral flow.

The numerous different scores and the dependence on acquisition techniques as well as individual parameters such as blood pressure or cardiac ejection fraction all can lead to considerable interrater variability in assessing collateral flow on CTA images and standardisation would be beneficial [48]. So far, there are only few reports on automated analysis of CTA images in acute stroke. Regarding automated analysis of collateral flow using CTA images, Boers and colleagues [49] used data from the MR CLEAN trial and compared automatically assessed quantitative CS with those from the trial’s core lab. They found a slightly but non-significantly better outcome prediction by automated CS than by manual scores.

There is a commercial software available from Brainomix called e-CTA® which is embedded in the company’s e-Stroke Suite platform. It determines the Tan score and also gives a percentage of vasculature detected in the affected MCA territory as compared to the contralateral side, similar to the aforementioned approach [49]. It has been evaluated first in the CATS study [29]. Here it was used to score single phase CTA images of 98 acute stroke patients with LVO. The automated CS achieved a high agreement with the consensus score from three experienced neuroradiologists and knowledge of the automated CS also significantly increased the interrater agreement between the three experts. In another study, using single phase CTAs from 235 acute stroke patients undergoing MT, CS from e-CTA were compared with those from two blinded neuroradiologists against an expert-based ground truth. Here, e-CTA again reached a similar level of agreement with ground truth as the professionals [50].

### Large vessel occlusion

Another application of machine learning algorithms to acute LVO CTA is automated detection of the occlusion. In contrast to CS, where automatization can overcome interrater variability, the advantage is different from LVO detection, since experts are not really challenged. However, less experienced physicians in primary care institutions will depend on the correct detection of an LVO when deciding to refer a patient to a comprehensive stroke centre or not. Technically, such an approach is straight forward as algorithms such as the “region growing” approach can be employed here. There are two reports on this issue so far. In the ALADIN study, Barreira and colleagues used a commercial software employing an AI-based algorithm (Viz LVO, Viz.ai, San Francisco, USA), in 875 patients with 46% LVO and compared the results against an expert-based ground truth [51, 52]. They report an accuracy of 86%, sensitivity of 90.1% & specificity of 82.5 for the software which took under 5 min. Seker et al. [53] used a machine learning algorithm developed by Brainomix in 144 acute stroke patients of whom 73 had an LVO and MT. Here, the algorithm reached accuracy, sensitivity and specificity of 90, 91 and 90%, respectively, when compared against ground truth. The average duration for each analysis was below 1 min. Furthermore, this performance was similar to that of two blinded neuroradiologists.

Automated analysis of CTA using AI-based algorithms can have several advantages in acute stroke imaging: i) interrater variability can be reduced to increase objectivity, ii) less experienced physicians can get support in diagnosing LVO and collateral flow and iii) decision making in stroke treatment can be accelerated. As with human reading, however, timing of the arrival of contrast is pivotal here, too.

## Perfusion imaging

Perfusion imaging can be performed with either CT or MRI. Whereas NCCT can only identify early ischemic changes, which – if present – are generally not reversible and turn into final infarction, perfusion imaging, like DWI has the chance to estimate the ischemic core even before ischemic changes can be seen on NCCT, in the very early time window. Furthermore perfusion imaging can visualize tissue at risk that might be rescuable. Perfusion imaging is a depiction of the passage of blood or fluids through the vessels of an organ or tissue to allow quantification. It enables visualisation of regions of abnormal cerebral hemodynamics and quantifies the effect of interfering situations, such as a preceding stenosis or an occlusion. The main principle of contrast-enhanced perfusion imaging is to monitor the first pass of a bolus of contrast agent through the cerebral circulation.

### Perfusion imaging parameters

In order to derive information from perfusion maps, it is important to define specific imaging parameters. Cerebral blood volume (CBV) is commonly referred to a volume of blood in a given region of brain tissue as per millilitres per 100 g of brain tissue [54]. Cerebral blood flow (CBF) is a certain amount of blood volume passing to a defined volume of brain tissue in a given period of time. This is usually expressed as millilitres of blood per minute per 100 g of brain tissue [54].

Mean transit time (MTT) is the average time (in seconds) a certain volume of blood takes to pass through a given volume of brain [54]. MTT is calculated by dividing CBV by CBF. Time to peak (TTP) is the time it takes an IV-injected bolus of contrast material to reach its peak in a given region of the brain, also commonly measured in seconds.

### Perfusion imaging pitfalls

Physicians need to be aware of major pitfalls that could lead to a falsified or useless perfusion imaging:

(i) The bolus needs to be recorded during the whole passage through the brain including the arterial, parenchymal and venous phase. As perfusion imaging acquisition usually takes only 40 to 60 s, a common pitfall occurs, for example, in patients with decreased cardiac output which leads to a delayed or slow increase in arterial input shifting the bolus curve to the end of imaging acquisition.
(ii) By placing the intravenous access very peripherally, for example in the back of the hand or foot of the patient, the contrast agent takes more time to reach the brain for imaging acquisition (see paragraph above) and the bolus gets diluted until it reaches the brain vessels. Therefore the increase in contrast agent is diminished, which leads to inaccurate perfusion maps, specifically to underestimated CBF and overestimated MTT, mimicking hypoperfusion.

In order to evaluate the quality of the perfusion data and to assess the reliability of the post-processing results, both situations need to be taken account for using the arterial input function (AIF) and venous output function (VOF) profiles during imaging post-processing. Computer-assisted imaging algorithms usually provide AIF and VOF profiles automatically. Moreover, “head shaking” head movement can be corrected for by using the midline of the brain. If, however, the patient moves his head within the longitudinal axis (in a figurative sense saying “yes”, nodding), the sections of the brain change the level or imaging slice and voxels usually cannot be reassigned properly during imaging postprocessing.

### Defining the ‘ischemic core’ and ‘tissue at risk’ using DWI, MR-perfusion (MRP) and CTP

Over the years, there have been several different approaches to distinguish normal tissue from hypoperfused, viable tissue (‘tissue at risk’) from nonviable tissue (‘ischemic core’) using CT- and MR-perfusion imaging. Correspondingly, numerous thresholds have been advocated [55]. Using CTP, the ischemic core is defined as a region with a substantial reduction in CBF with respect to the healthy contralateral hemisphere (rCBF). A substantial reduction is determined to be present as a reduced rCBF < 30% or 40%. DWI however, is recognised as a gold standard to assess the infarct core based on apparent diffusion coefficient (ADC) thresholds between < 0.6–0.62 × 10–3 mm2/s. (see the Additional file 1). For CTP and MR Perfusion, a delay in time to peak perfusion longer than 6 s (Tmax > 6 s) is considered to be a reliable predictor of the tissue at risk [56–59]. In a small patient cohort, Lin et al. could demonstrate that CTP and MR Perfusion could be used interchangeably if Tmax = 4 to 6 s measurements were used [60].

The amount of absolute (ml) or relative mismatch between ischemic core and tissue at risk varies within studies [61, 62], but a mismatch ratio of > 1.2 has been used in numerous prospective randomised controlled stroke trials (see the Additional file 1). However, other trials – DEFUSE 3, Solitaire™ FR With the Intention For Thrombectomy as Primary Endovascular Treatment for Acute Ischemic Stroke (SWIFT PRIME) trial – used a tighter mismatch ratio of ≥1.8 [63, 64]. Although the mismatch ratio originates from trials using MRP for patient selection, it is generally accepted to identify patients for reperfusion therapies based on CTP using an rCBF/Tmax mismatch ratio of > 1.2 [64, 65].

### Computerised assessment of perfusion imaging

Post-processing software estimates perfusion parameters based on above mentioned brain perfusion principles using deconvolution of tissue and arterial signals (e.g. singular value decomposition (SVD) deconvolution method, methods using Bayes theorem) in perfusion imaging (incl. co-registration with DWI images in MRI) with specified thresholds for an automated segmentation and quantification of the infarct core and the tissue at risk [66, 67]. For MRI, in order to reduce false-positive detection of ADC lesions of otherwise healthy tissue automated, infarct core volumetry within the tissue at risk can be used [68].

Because of different post-processing algorithms and thresholds – results differ among commercially available fully automated software even when using identical source data [69, 70]. Physicians need to be aware of this circumstance, especially in difficult decision-making situations or when treating acute stroke patients and perfusion results are somewhere in between against or in favour of a specific treatment. Yet, automated analysis seems to outperform human thresholding and analysis of CTP data [71].

Recently, a different approach has been used to either improve or replace previous software solutions. By using machine learning methods, i.e. deep learning techniques using neural networks, the infarct core volume and tissue at risk can be predicted directly from the CTP or MRP source images. Additional metadata – such as the time parameters and treatment – could further increase prediction accuracy and might even predict infarct growth over time or depending on treatment modality (see the Additional file 1).

### Perfusion imaging softwares in clinical trials

There are several vendors, respectively software products for post-processing of CT and MR perfusion images available; some of them are listed in Table 1. Since there are so many software solutions, it is difficult to list all of them and their validation within smaller studies or trials. Vendor-specific software usually has the advantage that thresholds can be chosen at the discretion of the radiologist evaluating the images. This, however, makes it prone to interrater variability leaving quantitative values – if provided by the software – hardly comparable.

The RApid processing of PerfusIon and Diffusion [RAPID] software (iSchemaView) – a commercial software solution – has been used and is currently used in a number of large stroke trials (see Table 2). However, prominent trials using RAPID – EXTEND-IA, DEFUS 3 and DAWN and DEFUSE 3 – only included 70, 182, respectively 206 patients. Furthermore, all these trials did not evaluate the performance of the software. As there are no studies or trials directly comparing their impact on patient outcome depending on the specific imaging post-processing results, it remains unclear whether available software solutions are interchangeable. Such trials, allowing inclusion of patients by multiple vendor products appear to be mandatory to further improve imaging evaluation and make its results more generalizable.

**Table 2:** A brief list of actively recruiting or completed large clinical trials using RApid processing of PerfusIon and Diffusion [RAPID] software (iSchemaView, Menlo Park, USA) for assessment of CT- and MRI- perfusion images

Trial Name	RAPID	Number of Patients	Status	Reference
DWI or CTP Assessment with Clinical Mismatch in the Triage of Wake-Up and Late Presenting Strokes Undergoing Neurointervention with Trevo (DAWN)	imaging selection for 100% of patients	206	Completed	[72]
The diffusion and perfusion imaging evaluation for understanding stroke evolution (DEFUSE) study	imaging selection for 100% of patients	74	Completed	[61]
The diffusion and perfusion imaging evaluation for understanding stroke evolution 3(DEFUSE 3) study	imaging selection for 100% of patients	182	Completed	[38]
Extending the Time for Thrombolysis in Emergency Neurological Deficits (International) (EXTEND)	imaging selection for 100% of patients	225	Completed	[73]
Extending the Time for Thrombolysis in Emergency Neurological Deficits - Intra-Arterial (EXTEND-IA)	imaging selection for 100% of patients	70	Completed	[74]
FRench Acute Cerebral Multimodal Imaging to Select Patient for MEchanical Thrombectomy (FRAME)	imaging selection for 100% of patients	Estimated Enrollment: 220 participants	Enrolling	ClinicalTrials.gov Identifier: NCT03045146
Solitaire™ With the Intention For Thrombectomy as PRIMary Endovascular Treatment (SWIFT PRIME) Trial	imaging selection for 100% of patients	196	Completed	[75]

## Conclusion

Automated image analysis of ischemic stroke with the support of machine learning or artificial intelligence related algorithms is a constantly growing market. Commercially and non-commercially available CAD products so far focus on the analysis of NCCT, CTA and perfusion imaging, based on CT or MR imaging. They aim to identify and quantify the ischemic core, the ischemic penumbra, the status of collateral flow and the site of arterial occlusion in an automatic fashion.

CAD algorithms are not intended as standalone diagnostic tools, however, they assist physicians to get more accurate and standardised interpretations of stroke related findings, which may improve the stroke management and patients’ selection for appropriate (usually time critical) treatments.

Future clinical studies are necessary for proper validation, evaluation and comparison of the different available software solutions in order to broaden and generalise treatment selection criteria for patients with acute ischemic stroke. Furthermore, future studies may focus on the integration of CAD algorithms within the workflow of stroke referral networks.

## Additional file

Additional file 1: Supplementary Appendix. (DOCX 102 kb)

## Data Availability

Not applicable.

## Abbreviations

AAPM: American Association of Physicists in Medicine
ACD: Automated computer diagnosis
ADC: Apparent diffusion coefficient
AI: Artificial intelligence
AIF: Arterial input function
ASPECTS: Alberta Stroke Programme Early CT Score
CA: Contrast agent
CAD: Computer-aided diagnosis
CADSC: Computer Aided Detection in Diagnostic Imaging Subcommittee
CAST: computer-aided simple triage
CBF: Cerebral blood flow
CBV: Cerebral blood volume
CE: European conformity
CS: Collateral score
CT: Computed tomography
CTA: Computed tomography angiography
CTP: Computed tomography perfusion
DL: Deep learning
DSA: Digital subtraction angiography
DWI: Diffusion-weighted magnetic resonance imaging
FDA: Food and drug administration
HDVS: Hyperdense vessel sign
HU: Hounsfield units
i.v.: Intravenous
ICA: Internal carotid artery
IMDRF: International Medical Device Regulators Forum
LVO: Large vessel occlusion
MCA: Middle cerebral artery
MRA: Magnetic resonance imaging angiography
MRI: Magnetic resonance imaging
MRP: Magnetic resonance imaging perfusion
mRS: Modified Ranking Scale
MT: Mechanical thrombectomy
mTICI: Modified treatment in cerebral infarction
MTT: Mean transit time
NCCT: Non-contrast-enhanced computed tomography
NIHSS: NIH Stroke Scale
rCBF: Substantial reduction in cerebral blood flow
RL: Representation learning
ROI: Regions of interest
SaMD: Software as a Medical Device
SVD: Singular value decomposition
TOAST: Trial of Org 10,172 in Acute Stroke Treatment
TTP: Time to peak
VOF: Venous output function
