# Supplementary material for: Computer-aided imaging analysis in acute ischemic stroke – background and clinical applications
Source: Neurol Res Pract. 2019 Aug 15;1:23. doi: 10.1186/s42466-019-0028-y (PMC7650084; doi:10.1186/s42466-019-0028-y)
Supplement: Supplementary file 1 — Supplementary Appendix. (DOCX 102 kb) [file 42466_2019_28_MOESM1_ESM.docx]

Supplementary Appendix

| Related topics | References |
| --- | --- |
| Publications presenting computer-aided diagnosis application in different medical domains | [1-8] |
| Image segmentation helps defining boundaries, objects or ROIs and reduces the complexity for the CAD algorithms during the further analysis In pixel-level, segmentation could be also seen as a classification process, in which segmentation algorithms define whether a pixel belongs to the background or to a target class | [9, 10] |
| The probably first semi-automated approach to identify putative hypodensity within middle cerebral artery (MCA) territory | [11] |
| Different computer aided detection schemes for cerebral ischemia on CT | [12-15] |
| A wavelet-based perception enhancement of the subtlest signs of hypodensity on computerized tomography exams | [16] |
| ASPECTS scores are have been shown to be a strong predictor of functional outcome and symptomatic intracerebral haemorrhage, following thrombolytic treatment and most importantly have been used as inclusion criteria for several thrombectomy trials (i.e. ESCAPE and ESCAPE-NA1, REVASCAT). | [17-19] |
| Performance studies or reports of the clinical application for e-ASPECTS within different settings and patients populations | [20-27] |
| Publications about different locations of HDVS | [28-30] |
| Prediction of infarct core volume and tissue at risk directly from the CTP or MRP source images using artificial intelligence methods (i. a. machine learning, deep learning) | [31-37] |
| Prospective randomized controlled stroke trials using mismatch ratio of > 1.2 | [38-41] |
| Use of CBF for determination of ischemic core in CTP | [42-44] |
| Use of Diffusion-weighted imaging (DWI) for infarct core assessment | [45-48] |
| Use of multiphase CTA perfusion for identifying ischemic core | [49] |

1. Doi, K., *Computer-aided diagnosis in digital chest radiography.* Advances in Digital Radiography: RSNA Categorical Course in Diagnostic Radiology Physics Syllabus, Oak Brooks, RSNA, 2003: p. 227–236.

2. Gilbert, F.J., et al., *Single reading with computer-aided detection for screening mammography.* N Engl J Med, 2008. **359**(16): p. 1675-84.

3. Lodwick, G., et al., *Computer diagnosis of primary bone tumor.* Radiology, 1963. **80**: p. 273-275.

4. Yoshida, H. and A.H. Dachman, *Computer-aided diagnosis for CT colonography.* Semin Ultrasound CT MR, 2004. **25**(5): p. 419-31.

5. Kuang, H., et al., *Automated ASPECTS on Noncontrast CT Scans in Patients with Acute Ischemic Stroke Using Machine Learning.* AJNR Am J Neuroradiol, 2019. **40**(1): p. 33-38.

6. Chilamkurthy, S., et al., *Deep learning algorithms for detection of critical findings in head CT scans: a retrospective study.* Lancet, 2018. **392**(10162): p. 2388-2396.

7. Hipp, J., et al., *Computer aided diagnostic tools aim to empower rather than replace pathologists: Lessons learned from computational chess.* J Pathol Inform, 2011. **2**: p. 25.

8. Lemke, H.U. and A. Melzer, *Back to the roots of AI and their relevance for health care today.* Minim Invasive Ther Allied Technol, 2019. **28**(2): p. 65-68.

9. Arimura, H., T. Magome, and Y. Yamashita, *Computer-aided diagnosis systems for brain diseases in magnetic resonance images.* Algorithms, 2009. **2**(3): p. 925-952.

10. Chartrand, G., et al., *Deep Learning: A Primer for Radiologists.* Radiographics, 2017. **37**(7): p. 2113-2131.

11. Maldjian, J.A., et al., *Automated CT segmentation and analysis for acute middle cerebral artery stroke.* AJNR Am J Neuroradiol, 2001. **22**(6): p. 1050-5.

12. Hema Rajini, N. and R. Bhavani, *Computer aided detection of ischemic stroke using segmentation and texture features.* Measurement, 2013. **46**(6): p. 1865-1874.

13. Saito, H., et al., *[A computerized method for detection of acute cerebral infarction on CT images].* Nihon Hoshasen Gijutsu Gakkai Zasshi, 2010. **66**(9): p. 1169-77.

14. Takahashi, N., et al., *[Development of an algorithm for the detection of early signs of cerebral ischemia on CT images].* Nihon Hoshasen Gijutsu Gakkai Zasshi, 2007. **63**(8): p. 835-42.

15. Tang, F.H., D.K. Ng, and D.H. Chow, *An image feature approach for computer-aided detection of ischemic stroke.* Comput Biol Med, 2011. **41**(7): p. 529-36.

16. Przelaskowski, A., et al., *Improved early stroke detection: wavelet-based perception enhancement of computerized tomography exams.* Comput Biol Med, 2007. **37**(4): p. 524-33.

17. Barber, P.A., et al., *Validity and reliability of a quantitative computed tomography score in predicting outcome of hyperacute stroke before thrombolytic therapy. ASPECTS Study Group. Alberta Stroke Programme Early CT Score.* Lancet, 2000. **355**(9216): p. 1670-4.

18. Hill, M.D., et al., *Selection of acute ischemic stroke patients for intra-arterial thrombolysis with pro-urokinase by using ASPECTS.* Stroke, 2003. **34**(8): p. 1925-31.

19. Okazaki, S., et al., *Extremely early computed tomography signs in hyperacute ischemic stroke as a predictor of parenchymal hematoma.* Cerebrovasc Dis, 2008. **25**(3): p. 241-6.

20. Demeestere, J., et al., *Alberta Stroke Program Early CT Score Versus Computed Tomographic Perfusion to Predict Functional Outcome After Successful Reperfusion in Acute Ischemic Stroke.* Stroke, 2018. **49**(10): p. 2361-2367.

21. Grunwald, I.Q., et al., *First Automated Stroke Imaging Evaluation via Electronic Alberta Stroke Program Early CT Score in a Mobile Stroke Unit.* Cerebrovasc Dis, 2016. **42**(5-6): p. 332-338.

22. Guberina, N., et al., *Detection of early infarction signs with machine learning-based diagnosis by means of the Alberta Stroke Program Early CT score (ASPECTS) in the clinical routine.* Neuroradiology, 2018. **60**(9): p. 889-901.

23. Herweh, C., et al., *Performance of e-ASPECTS software in comparison to that of stroke physicians on assessing CT scans of acute ischemic stroke patients.* Int J Stroke, 2016. **11**(4): p. 438-45.

24. Nagel, S., et al., *e-ASPECTS software is non-inferior to neuroradiologists in applying the ASPECT score to computed tomography scans of acute ischemic stroke patients.* Int J Stroke, 2017. **12**(6): p. 615-622.

25. Nagel, S., et al., *Clinical Utility of Electronic Alberta Stroke Program Early Computed Tomography Score Software in the ENCHANTED Trial Database.* Stroke, 2018. **49**(6): p. 1407-1411.

26. Olive-Gadea, M., et al., *Baseline ASPECTS and e-ASPECTS Correlation with Infarct Volume and Functional Outcome in Patients Undergoing Mechanical Thrombectomy.* J Neuroimaging, 2018.

27. Pfaff, J., et al., *e-ASPECTS Correlates with and Is Predictive of Outcome after Mechanical Thrombectomy.* AJNR Am J Neuroradiol, 2017. **38**(8): p. 1594-1599.

28. Vogler, J.t., et al., *Bilateral hyperdense middle cerebral arteries: Stroke sign or not?* Radiol Case Rep, 2018. **13**(5): p. 933-935.

29. Krings, T., et al., *The hyperdense posterior cerebral artery sign: a computed tomography marker of acute ischemia in the posterior cerebral artery territory.* Stroke, 2006. **37**(2): p. 399-403.

30. Ernst, M., et al., *Sensitivity of Hyperdense Basilar Artery Sign on Non-Enhanced Computed Tomography.* PLoS One, 2015. **10**(10): p. e0141096.

31. Feng, R., et al., *Deep learning guided stroke management: a review of clinical applications.* J Neurointerv Surg, 2018. **10**(4): p. 358-362.

32. McKinley, R., et al., *A Machine Learning Approach to Perfusion Imaging With Dynamic Susceptibility Contrast MR.* Front Neurol, 2018. **9**: p. 717.

33. Kamal, H., V. Lopez, and S.A. Sheth, *Machine Learning in Acute Ischemic Stroke Neuroimaging.* Front Neurol, 2018. **9**: p. 945.

34. Vargas, J., A. Spiotta, and A.R. Chatterjee, *Initial Experiences with Artificial Neural Networks in the Detection of Computed Tomography Perfusion Deficits.* World Neurosurg, 2018.

35. Tang, T.Y., et al., *Development and validation of a penumbra-based predictive model for thrombolysis outcome in acute ischemic stroke patients.* EBioMedicine, 2018. **35**: p. 251-259.

36. Lucas, C., et al., *Learning to Predict Ischemic Stroke Growth on Acute CT Perfusion Data by Interpolating Low-Dimensional Shape Representations.* Front Neurol, 2018. **9**: p. 989.

37. Robben, D., et al., *Prediction of final infarct volume from native CT perfusion and treatment parameters using deep learning*. 2018.

38. Ma, H., et al., *A Multicentre, Randomized, Double-Blinded, Placebo-Controlled Phase III Study to Investigate Extending the Time for Thrombolysis in Emergency Neurological Deficits (EXTEND).* 2012. **7**(1): p. 74-80.

39. Campbell, B.C.V., et al., *Endovascular Therapy for Ischemic Stroke with Perfusion-Imaging Selection.* 2015. **372**(11): p. 1009-1018.

40. Amiri, H., et al., *European Cooperative Acute Stroke Study-4: Extending the time for thrombolysis in emergency neurological deficits ECASS-4: ExTEND.* Int J Stroke, 2016. **11**(2): p. 260-7.

41. Davis, S.M., et al., *Effects of alteplase beyond 3 h after stroke in the Echoplanar Imaging Thrombolytic Evaluation Trial (EPITHET): a placebo-controlled randomised trial.* Lancet Neurol, 2008. **7**(4): p. 299-309.

42. Bivard, A., et al., *Defining the extent of irreversible brain ischemia using perfusion computed tomography.* Cerebrovasc Dis, 2011. **31**(3): p. 238-45.

43. Kamalian, S., et al., *CT cerebral blood flow maps optimally correlate with admission diffusion-weighted imaging in acute stroke but thresholds vary by postprocessing platform.* Stroke, 2011. **42**(7): p. 1923-8.

44. Campbell, B.C., et al., *Cerebral blood flow is the optimal CT perfusion parameter for assessing infarct core.* Stroke, 2011. **42**(12): p. 3435-40.

45. Purushotham, A., et al., *Apparent Diffusion Coefficient Threshold for Delineation of Ischemic Core.* 2015. **10**(3): p. 348-353.

46. Lansberg, M.G., et al., *MRI profile and response to endovascular reperfusion after stroke (DEFUSE 2): a prospective cohort study.* Lancet Neurol, 2012. **11**(10): p. 860-7.

47. Nael, K., et al., *Multiparametric Magnetic Resonance Imaging for Prediction of Parenchymal Hemorrhage in Acute Ischemic Stroke After Reperfusion Therapy.* Stroke, 2017. **48**(3): p. 664-670.

48. Xie, Y., et al., *Pretreatment lesional volume impacts clinical outcome and thrombectomy efficacy.* Ann Neurol, 2018. **83**(1): p. 178-185.

49. Reid, M., et al., *Accuracy and Reliability of Multiphase CTA Perfusion for Identifying Ischemic Core.* Clin Neuroradiol, 2018.
